# Body composition and CO_2_ dietary emissions

**DOI:** 10.3389/fpubh.2024.1432109

**Published:** 2025-01-17

**Authors:** Silvia García, Margalida Monserrat-Mesquida, Sebastián Mas-Fontao, Esther Cuadrado-Soto, María Ortiz-Ramos, Pilar Matía-Martín, Lidia Daimiel, Clotilde Vázquez, Josep A. Tur, Cristina Bouzas

**Affiliations:** ^1^CIBER Fisiopatología de la Obesidad y Nutrición (CIBEROBN), Instituto de Salud Carlos III (ISCIII), Madrid, Spain; ^2^Research Group on Community Nutrition & Oxidative Stress, University of Balearic Islands-IUNICS, Palma de Mallorca, Spain; ^3^Health Research Institute of the Balearic Islands (IdISBa), Palma de Mallorca, Spain; ^4^Department of Endocrinology and Nutrition, Hospital Fundación Jimenez Díaz, Instituto de Investigaciones Biomédicas IISFJD, University Autonóma de Madrid (UAM), Madrid, Spain; ^5^Nutritional Control of the Epigenome Group, Precision Nutrition and Obesity Program, IMDEA Food, CEI UAM + CSIC, Madrid, Spain; ^6^Department of Endocrinology and Nutrition, Instituto de Investigación Sanitaria Hospital Clínico San Carlos (IdISSC), Madrid, Spain; ^7^Department of Medicine, University Complutense of Madrid, Madrid, Spain; ^8^Departament of Pharmaceutical and Health Sciences, Faculty of Pharmacy, University San Pablo-CEU, CEU Universities, Madrid, Spain

**Keywords:** anthropometry, body composition, environment, CO_2_ emissions, sustainable diets

## Abstract

**Background:**

The amount and quality of foods consumed not only impact on individual health, as reflected in body composition, but they could influence on greenhouse gas emissions and then, on environment.

**Aim:**

This study aims to assess the relationship between the body composition and the CO_2_ emissions resulting from the dietary choices of an adult population.

**Design:**

A cross-sectional study on baseline data from 778 participants aged 55–75 years old, with metabolic syndrome (MetS) as part of the PREDIMED-Plus study.

**Methods:**

Food intake was registered using a validated semi quantitative 143-item food frequency questionnaire. The amount of CO_2_ emitted was calculated using data from the Agribalyse® 3.0.1 database. Anthropometry (body weight, height, and waist, and hip circumference, and body mass index) was determined by usual measurements, and body composition (fat mass, visceral fat, muscular mass, fat free mass, and total body water) were assessed by bioimpedance.

**Results:**

CO_2_ emissions were linearly and positively associated with weight, waist circumference, visceral fat, fat free mass, total body water and energy intake.

**Conclusion:**

Body composition is associated with dietary CO_2_ emissions. The higher total body water, fat free mass, and body weight, the higher the dietary CO_2_ emissions were, following a linear relationship.

**Clinical trial registration:**

http://www.isrctn.com/ISRCTN89898870, ISRCTN89898870.

## 1 Introduction

The increased greenhouse gas emissions (GHGEs) mostly contribute to the climate change and global warming, which are the main effects resulting from this GHGEs increase in the atmosphere, specifically from the rise in carbon dioxide (CO_2_), which is the main contributor (1). A significant portion of the generation of these GHGEs is of human origin. The human metabolic activity contributes to GHGEs by two ways: respiration and food intake. First, respiration cyclically involves the intake of oxygen (inspiration) and the release of carbon dioxide (expiration). Oxygen intake is used in oxidation reactions that release energy, yielding carbon dioxide CO_2_. Second, the energy expenditure of a human being on performing a specific task is compensated by a food intake of proportional energy (2). The choices we make when selecting the type of food to meet our energy needs have a very significant impact on the environment and the CO_2_ emissions generated, as the current food system contributes to this environmental impact (3–5). The food choices we make also affect our health and body shape (6). Therefore, it would be interesting to explore how the diet can contribute in a dual sense to both the health of the population and environmental well-being.

To identify diets that support individual health and environmental sustainability is the first step in developing strategies to promote sustainable consumer behaviors (3). Previous findings pointed out that around 16% of the US population changed their diets to align with recommendations for environmental sustainability (7). Diets consumed by Spanish (8), British (9), or Lebanese (10) people showed that GHGEs was lower in diets following the Mediterranean-style diet. The same was described for British people following the DASH diet (10, 11).

In addition to the relationship between health and diet, there is a special connection between body composition and diet. Body composition refers to the proportion of fat, muscle, bone, and other tissues that make up the body. It provides valuable insights into individual’s health status, offering a more precise understanding of physical condition than body weight alone. The connection between dietary intake and body composition is evident in how diet can affect energy consumption, acquired nutrients, and consequently, the distribution of fat and muscle mass in the body (12). This interrelation, in turn, is connected to environmental impact, as dietary choices influence food production and distribution, contributing to GHGEs (13).

The assessment of body composition emerges as a crucial aspect in evaluating nutritional status, providing pertinent data for detecting potential nutrition-related diseases and evaluating nutritional interventions (12). Anthropometry is based in non-invasive quantitative body measurements such as height, weight, head circumference, body mass index (BMI), body circumferences (waist, hip, and limps), and skinfold thickness (14). Body compartments such as fat, bone and muscle mass can be predicted from these anthropometric measurements (15).

Body composition measures may be inherently linked to environmental impact due to the direct influence of dietary choices and consumption patterns on both aspects (16). The amount and quality of foods consumed not only impact on individual health, as reflected in body composition, but also, they could exert substantial influence on GHGEs and, consequently, on environmental impact. This adverse situation could be diminished by changing dietary habits (17). The adoption of more sustainable diets can not only promote a healthy body composition, but they may be also associated with lower CO_2_ emissions (18–21).

Understanding and exploring the connection between body composition and environmental impact can shed light on the importance of adopting sustainable dietary practices for both individual health and environmental preservation. To fill this gap in the current literature, this current study aims to assess the relationship between the body composition and the CO_2_ emissions resulting from the dietary choices of an adult population.

## 2 Methods

### 2.1 Design

The current study was a cross-sectional analysis carried out on several baseline participants of the PREDIMED-PLUS trial, an eight-year, parallel-group, randomized trial conducted in several regions in Spain which aimed to see the effect of an energy-restricted traditional Mediterranean Diet combined with physical activity on cardiovascular disease morbimortality. Specific information related to the study protocol can be found elsewhere (22) and at http://predimedplus.com/. The trial was registered by The International Standard Randomized Controlled Trial (ISRCT)^1^ with the number 89898870 in 2014.

### 2.2 Participants, recruitment, and ethics

**Figure 1:**
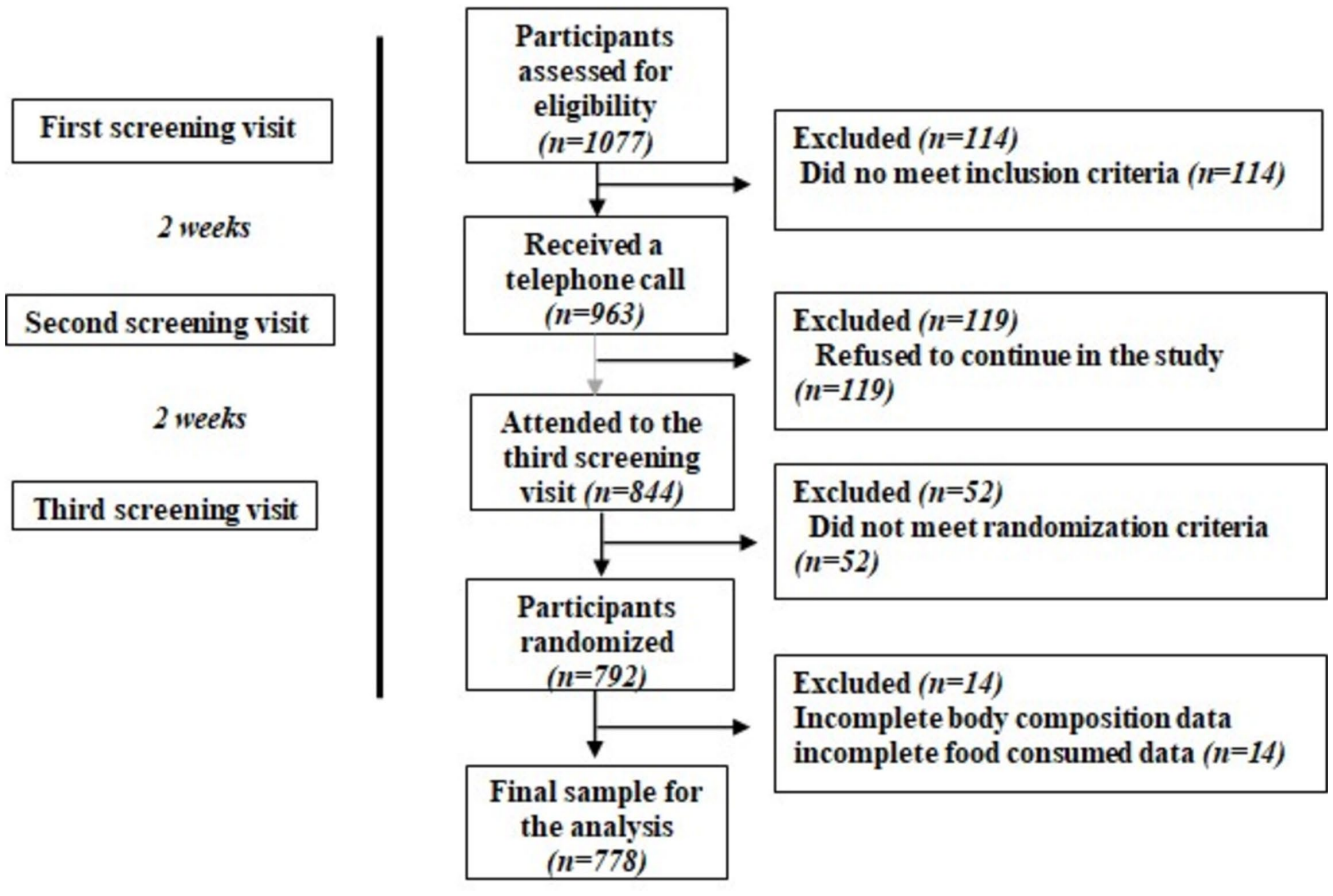
Flow chart of eligibility of participants.

Inclusion criteria of participants were to be 55–75-year-old, to have a body mass index (BMI) 27–40 kg/m^2^ and had to meet three or more criteria of the metabolic syndrome according to the International Diabetes Federation and the American Heart Association/National Heart, Lung, and Blood Institute (23). A number of 1,077 participants were initially assessed for eligibility. The final analysis in the present study was done with 778 participants, which had complete data on body composition, and on food consumption. Figure 1 shows the eligible participant’s flow-chart.

Ethical committees based on the ethical standards of the Declaration of Helsinki approved the study protocol for all the participating institutions, including the approval from the Ethics Committee of the Balearic Islands (ref. IB 2251/14 PI; Feb 26, 2014). All participants provided an informed written consent before participation.

### 2.3 Assessment of dietary intake

To assess the food consumed and the usual dietary intake of participants, a validated semi quantitative 143-item food frequency questionnaire (24–26) was administered by trained dietitians to assess usual dietary intakes of participants. Energy and nutrient intakes were calculated using a computer program based on available information from Spanish food composition tables (27, 28). The results determined the amount of food (in grams) and the energy intake (in kcal) consumed for each participant per day.

### 2.4 Calculation of CO_2_ emissions

Agribalyse® 3.0.1 database was used to calculate GHGEs. It was developed collaboratively by the French Agency for the Environment and Energy Management (ADEME) in conjunction with the CIQUAL French food composition table (29, 30). Ecoinvent® also cooperated with Agribalyse® 3.0.1, contributing with data for non-agricultural processes such as electricity and transport, as well as imported production data. This joint effort aimed to capture the production and market conditions prevalent in European countries. The project started in 2009, with the database being officially published in 2021.

The Agribalyse® 3.0.1 database serves as a valuable resource for reference data on the environmental impacts associated with agricultural and food products. It follows a comprehensive Life Cycle Assessment (LCA) methodology, breaking down the food chain into distinct stages. These stages encompass agricultural production, transportation, processing, packaging, distribution, retailing, consumer preparation, and disposal of packaging, with the exclusion of home wastage and transport from retail to households. The methodology adheres to internationally recognized LCA standards, including ISO 14040 and ISO 14044 (31, 32), LEAP guidelines (33), and the product environmental footprint (PEF) framework (34). Environmental indicators are reported per kilogram of product, yielding a total of 14 indicators with a single-score environmental footprint.

The amount of GHGEs from the dietary intake was calculated according to calculated grams of food consumed. GHGEs were calculated in terms of kg of carbon dioxide equivalents (CO_2_eq), applying the following formula for each specific food item:

GHGEs = ((grams of each reported food) x (kg of CO_2_eq emitted for that specific food)) / (1,000 grams of the corresponding food)

Subsequently, the sum of the total CO_2_eq emissions for the entire diet was determined.

### 2.5 Sociodemographic characteristics

Sociodemographic characteristics information such as sex, age, and educational level (primary school, secondary school, college school technician or bachelor’s degree) were self-reported by participants.

### 2.6 Anthropometric and body composition measurements

Anthropometric measurements were obtained, and BMI was calculated with the standard formula: Weight in kilograms divided by the square of height in meters (kg/m^2^). Registered dieticians measured height two-times with the participant’s head maintained in the Frankfurt Horizontal Plane using a wall-mounted stadiometer (Seca 213, HealthCheck Systems, Brooklyn, NY). Both waist and hip circumference were measured twice with an anthropometric tape, the average value of each measurement was used in the analysis. Waist circumference was measured halfway between the last rib and the iliac crest and hip circumference was measured around the widest part of the hip.

Body weight (kg), fat mass (kg), muscular mass (kg), fat free mass (kg) total body water (kg), and visceral fat rating (being 13 the cut-off point between low and high values) were assessed using a Segmental Body Composition Analyzer for impedance testing, BIA (Tanita MC780P-MA P, Tanita, Tokyo, Japan). Percentages were also calculated for all measures except for visceral fat, which was assessed in absolute terms. To measure body composition, participants needed to be in a standing position and wear light clothes and no shoes (0.6 kg was subtracted for their clothing). The BIA is based on the application of a weak electrical current through the human body to characterize the conductive and nonconductive tissue and fluid components of the body. The applied current flows in different rates depending on the body composition: It is well-conducted by water and electrolyte-rich tissues (blood and muscle) but is poorly conducted by fat, bone, and air-filled spaces (35). Fat mass, muscular mass, fat free mass and total body water measurements were calculated in kg and then divided by the squared size (m^2^) to adjust by height.

### 2.7 Statistics

SPSS statistical software package version 27.0 (SPPS Inc., Chicago, IL, USA) was used to perform the analysis. The quantity of CO_2_eq emitted was separated in quintiles from the group that emitted the lowest emissions to that emitted the highest: Quintile 1 (Q1); ≤4.3 kg CO_2_, quintile 2 (Q2); 4.4–5.1 kg CO_2_, quintile 3 (Q3); 5.2–5.8 kg CO_2_, quintile 4 (Q4); 5.9–6.7 kg CO_2_ and quintile 5 (Q5); >6.7 kg CO_2_. Prevalence data was expressed as simple size and percentage. Data was shown as mean and standard deviation (SD) for continuous variables and UNIANOVA was calculated adjusted by sex for anthropometric and body composition variables. To measure linear correlation, Pearson correlation was calculated between quintiles of CO_2_ emissions and anthropometric and body composition variables. Linear regression analysis was done for those variables that showed significance on the Pearson analysis and scatter plot graphics were also presented.

## 3 Results

Characteristics of the sample are showed in Table 1. Table 2 shows parameters of body composition distributed by sex according to CO_2_ emissions distributed in quintiles, and presented as the whole sample, and by sex. Body weight, waist circumference, hip circumference, fat mass (in kg), fat free mass (in kg), total body water (in kg), and visceral fat were directly associated to CO_2_ emissions quintiles. Table 2 also shows a significative correlation between energy intake and CO_2_ emissions. The higher the body composition values mentioned and energy intake, the higher the CO_2_ emissions were.

**Table 1:** Characteristics of the sample.

		*n* (%)
Total		778
Sex	Men	418 (53.7)
	Women	360 (46.3)
Highest school level completed	Bachelor’s degree	187 (24.0)
	College School Technician	55 (7.1)
	Secondary School	270 (34.7)
	Primary School	266 (34.2)
		Mean (±SD)
	Age (yr)	64.6 (5.2)
	Body weight (kg)	88.2 (13.4)
	BMI (kg/m^2^)	32.8 (3.5)
	Energy intake (Kcal/day)	2,477 (746)

SD, Standard deviation; BMI, Body Mass Index.

**Table 2:** Body composition measurements distributed by sex according to CO_2_ emissions (quintiles).

	Q1 = 155 (<4.3 kg CO_2_)	Q2 = 156 (4.4–5.1 kg CO_2_)	Q3 = 156 (5.2–5.8 kg CO_2_)	Q4 = 156 (5.9–6.7 kg CO_2_)	Q5 = 155 (>6.7 kg CO_2_)	*p*
Body weight (kg)	86.4 (13.8)	86.4 (12.7)	87.5 (13.2)	88.9 (13.2)	92.2 (13.2)	0.002
Men	94.1 (13.6)	93.3 (11.1)	94.8 (11.2)	94.5 (11.2)	96.2 (12.1)	
Women	81.3 (11.4)	78.6 (9.7)	80.1 (10.7)	80.8 (11.8)	84.1 (11.8)	
Waist circumference (cm)	108.9 (10.5)	108.3 (9.7)	108.8 (10.2)	109.1 (10.1)	112.1 (9.7)	0.002
Men	112.5 (9.3)	112.3 (8.8)	113.2 (9.6)	113.0 (9.1)	114.2 (9.2)	
Women	106.6 (10.5)	103.9 (8.7)	104.4 (8.8)	103.5 (8.5)	108.0 (9.4)	
Hip circumference (cm)	113.0 (9.6)	110.5 (8.7)	111.6 (9.1)	110.9 (8.1)	112.5 (9.4)	0.017
Men	109.6 (7.6)	108.6 (7.4)	110.1 (8.0)	109.3 (6.8)	110.5 (8.2)	
Women	115.2 (10.2)	112.5 (9.6)	113.1 (10.0)	113.3 (9.1)	116.6 (10.5)	
BMI (kg/m^2^)	33.4 (3.5)	32.4 (3.3)	32.7 (3.6)	32.5 (3.2)	33.2 (3.7)	0.053
Men	32.7 (3.3)	32.2 (3.0)	32.6 (3.4)	32.3 (3.0)	32.8 (3.6)	
Women	33.8 (3.6)	32.7 (3.7)	32.8 (3.8)	32.6 (3.4)	34.0 (3.8)	
Fat mass (kg)	32.7 (8.1)	32.1 (7.2)	32.7 (8.2)	31.7 (8.1)	33.0 (8.2)	0.003
Men	29.1 (7.2)	30.1 (7.0)	30.2 (7.5)	29.2 (6.9)	31.0 (7.4)	
Women	35.1 (7.8)	34.3 (6.7)	35.4 (8.0)	35.5 (8.2)	37.5 (8.3)	
Fat mass (%)	38.5 (7.6)	37.8 (7.4)	37.6 (7.8)	36.1 (7.9)	36.0 (7.6)	0.170
Men	31.2 (4.4)	32.2 (4.6)	31.7 (4.7)	31.0 (4.8)	32.0 (4.1)	
Women	43.5 (4.8)	43.8 (4.6)	43.8 (5.1)	43.9 (5.0)	45.1 (5.7)	
Muscular mass (kg)	37.3 (14.6)	37.0 (15.3)	36.6 (15.4)	38.8 (17.1)	34.1 (15.7)	0.411
Men	41.1 (16.4)	43.1 (16.0)	41.7 (15.9)	44.3 (17.4)	39.6 (15.4)	
Women	34.4 (12.3)	31.2 (12.1)	31.4 (13.0)	29.1 (11.5)	23.5 (9.8)	
Muscular mass (%)	42.6 (16.8)	38.5 (16.8)	37.8 (16.1)	36.7 (14.7)	35.7 (17.4)	0.169
Men	41.5 (17.0)	40.2 (17.5)	39.1 (15.9)	38.5 (14.5)	41.1 (18.2)	
Women	43.6 (16.7)	36.8 (16.1)	36.3 (16.4)	33.5 (14.7)	24.8 (8.8)	
Fat free mass (kg)	52.3 (11.1)	53.1 (11.2)	54.4 (10.9)	56.5 (11.7)	58.7 (11.3)	0.003
Men	63.4 (7.6)	62.3 (6.5)	63.7 (5.7)	64.2 (7.4)	64.6 (6.7)	
Women	44.8 (5.3)	43.2 (5.1)	44.6 (4.7)	44.7 (5.6)	45.1 (7.1)	
Fat free mass (%)	61.4 (7.6)	62.1 (7.6)	62.3 (7.8)	63.8 (7.9)	63.9 (7.6)	0.169
Men	68.9 (4.3)	67.7 (4.6)	68.2 (4.7)	68.8 (4.8)	67.9 (4.1)	
Women	56.4 (4.8)	55.9 (5.0)	56.1 (5.1)	56.1 (4.9)	54.8 (5.7)	
Total body water (kg)	36.7 (4.7)	35.3 (4.0)	40.6 (7.6)	44.7 (6.9)	44.6 (9.1)	0.006
Men	42.5 (4.0)	42.5 (1.7)	46.3 (2.2)	49.4 (3.5)	47.0 (6.8)	
Women	34.4 (2.5)	33.7 (2.1)	32.0 (1.1)	37.0 (3.1)	28.0 (0.0)	
Total body water (%)	45.1 (4.7)	46.1 (4.9)	46.1 (5.0)	46.1 (4.1)	47.8 (4.3)	0.079
Men	50.9 (2.5)	49.6 (3.7)	50.4 (2.8)	48.6 (2.3)	49.5 (3.1)	
Women	42.2 (2.4)	42.9 (3.5)	42.3 (3.1)	41.4 (1.8)	41.4 (1.9)	
Visceral fat (units)	15.2 (3.8)	15.7 (4.1)	15.7 (4.3)	15.8 (3.9)	16.9 (4.2)	0.034
Men	18.1 (3.3)	17.9 (3.5)	18.0 (3.8)	17.7 (3.1)	18.4 (3.9)	
Women	13.1 (2.5)	13.3 (3.4)	13.1 (3.2)	12.7 (3.0)	13.6 (2.3)	
Energy intake (kcal/day)	1843.9 (439.7)	2187.6 (433.6)	2374.7 (492.2)	2759.8 (547.1)	3219.8 (863.1)	<0.001
Men	1879 (455)	2,276 (436)	2,391 (511)	2,862 (545)	3,315 (940)	
Women	1821 (428)	2090 (410)	2,358 (473)	2,612 (516)	3,026 (641)	

Values are presented in mean (SD). BMI, Body Mass Index; CO_2_, Carbon dioxide. Q1: Quintile 1. Q2: Quintile 2. Q3: Quintile 3. Q4: Quintile 4. Q5: Quintile 5. SD, Standard deviation. Differences between groups were tested by UNIANOVA adjusted by sex.

The highest values of most of body composition parameters (body weight, waist circumference, hip circumference, fat mass, fat free mass, visceral fat, and energy intake) were found in the last quintile (highest CO_2_ emissions) in both sexes.

Table 3 shows correlations between quintiles of CO_2_ emissions and body composition. The correlation between energy intake and quintiles of CO_2_ emissions is also shown. Quintiles of CO_2_ emissions were linearly and positively correlated (*p* < 0.001) with weight (*r* = 0.149), waist circumference (*r* = 0.099), fat free mass (*r* = 0.198), total body water (*r* = 0.495), visceral fat (*r* = 0.118), and energy intake (*r* = 0.629).

**Table 3:** Pearson correlations between body composition, energy intake and CO_2_ emissions.

	*r*	*p*
Body weight (kg)	0.149	<0.001
Waist circumference (cm)	0.099	<0.001
Hip circumference (cm)	−0.007	0.772
BMI (kg/m^2^)	−0.016	0.541
Fat mass (kg)	0.000	0.997
Muscular mass (kg)	−0.040	0.267
Fat free mass (kg)	0.198	<0.001
Total body water (kg)	0.495	<0.001
Visceral fat (units)	0.118	<0.001
Energy intake (kcal/day)	0.629	<0.001

Person correlation was calculated between quintiles of CO_2_ emissions and anthropometric and body composition variables, and energy intake. BMI, Body Mass Index; CO_2_, Carbon dioxide; *r*, Correlation coefficient.

Table 4 shows *R* and *R*^2^ values of the linear analysis between total CO_2_ emissions and body composition. *R*^2^ values were 0.016 (weight), 0.005 (waist circumference), 0.030 (fat free mass), 0.226 (total body water), 0.011 (visceral fat) and 0.500 (energy intake), all with high significance level.

**Table 4:** Linear regression analysis between total CO_2_ emissions and body composition.

		Weight	Waist circumference	Fat free mass	Total body water	Visceral fat	Energy intake
Total CO_2_ emissions	*R*	0.125	0.074	0.173	0.476	0.105	0.707
	*R* ^2^	0.016	0.005	0.030	0.226	0.011	0.500
	*y*=	82.59–1.01*x	1.07E^2^ + 0.45*x	48.05 + 1.25*x	26.42 + 2.57*x	14.39 + 0.27*x	691 + 316*x
	*p*	<0.001	0.004	<0.001	<0.001	<0.001	<0.001

Linear regression analysis was done for Pearson significant variables. Correlation and determination coefficients were calculated, and regression line was showed. CO_2_, Carbon dioxide; R, Correlation coefficient; R2, Determination coefficient. *means multiplying.

**Figure 2:**
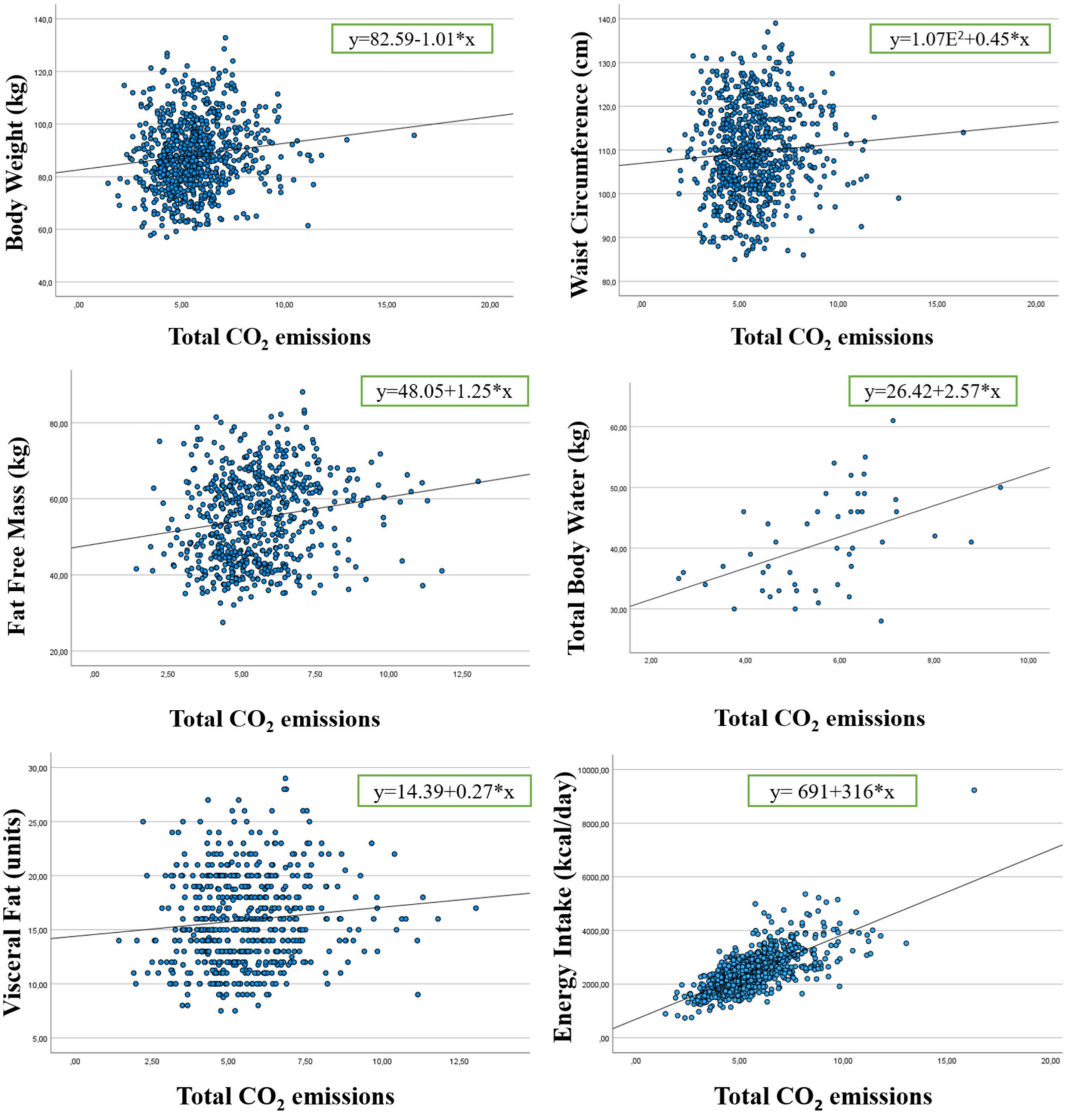
Scatter plot graphics and regression lines of the total CO_2_ emissions and weight, waist circumference, fat free mass, total body water, visceral fat, and energy intake.

Figure 2 shows scatter plot graphics and regression lines of the total CO_2_ emissions and weight, waist circumference, fat free mass, total body water, visceral fat, and energy intake. The data of all parameters analyzed show an uphill pattern from left to right, which indicates a positive linear relationship between X values (weight, waist circumference, fat free mass, total body water, visceral fat, and energy intake) and Y values (total CO_2_ emissions).

## 4 Discussion

The current study showed that individuals with higher energy intake, and correspondingly higher total body water (kg), fat free mass (kg), and body weight (kg), have higher dietary CO_2_ emissions than individuals with lower energy intake and smaller body size, following a linear relationship.

Body composition reflects the nutritional and health status (36, 37). Measuring body compartments, such as fat mass, visceral fat, and muscle, mass allows for a better diagnosis of nutritional status (38). The current results showed higher values of fat free mass and total body water as dietary CO_2_ emissions increased. Higher fat-free mass is advantageous with aging and is protective against sarcopenia (39), but only if this fat-free mass is attributable to muscle mass. Fat-free mass comprises muscle, organs, bones, and body water (40). The current results for muscular mass and fat-free mass showed trends in opposite directions across quintiles, which may seem contradictory. This discrepancy arises because, in the older adult population, data on fat-free mass can be misinterpreted due to increased water retention in older individuals (41), not due to an increase in muscle mass. It is important to note that the current findings showed how the increase in fat-free mass may be primarily attributed to the rise in total body water, rather than being attributed only to a rise in dietary CO_2_ emissions.

Body composition, referring to the distribution of tissues like fat and muscle, tends to vary between sexes due to biological and hormonal differences. Generally, women have a slightly higher body fat percentage, while men typically have more muscle mass. These variations, are influenced by genetic, hormonal, and metabolic factors, contribute to differences in appearance and body composition between men and women (42). When the current results were analyzed by sex, changes between groups vary slightly depending on it, and the overall trend remained consistent with the combined data for both sexes, except for total body water. In the case of women, total body water decreased as CO_2_ increased. This phenomenon seems to be attributed not to an increase of muscle mass but rather to the age-related increase in total body fat. The rise in subcutaneous fat accumulation is especially prevalent in women, while visceral fat tends to increase more in men (39).

Current findings also showed a close relationship between energy intake and dietary CO_2_ emissions. This underscores the significance of energy intake on both body composition and environmental impact, and it is consistent with previous studies showing that the relationship between diet quality and GHGEs becomes more apparent when considering energy intake (9). The impact of energy intake on dietary CO_2_ emissions was also considered in previous studies evaluating dietary characteristics (14, 43, 44). Our current research shows that as the dietary CO_2_ emissions rose, there was a corresponding increase in energy intake, which is aligned with an increase in body weight, visceral fat, and waist circumference.

The current findings on the association of body composition with the environmental impact of a diet is consistent with findings observed in other studies (45–47). The EAT-Lancet Reference Diet (ELR-diet) found inverse associations between higher adherence to ELR-diet and anthropometric markers such as body weight, waist circumference, BMI, fat free mass index, and body fat percentage. Higher ELR-diet adherence was also inversely associated with lower environmental impact, measured in GHGEs and land used (47). Another study identified that enhancing awareness and nutritional education could serve as a strategy to simultaneously improve both sustainability and anthropometry toward healthy values. The research revealed that as sustainable consumption behaviors and food literacy increased, there was a corresponding reduction in BMI, body weight, and waist-to-hip ratio (48). The observed decrease in visceral fat among current participants following a lower dietary CO_2_ may be attributed to a key characteristic of a sustainable diet: a decrease in the consumption of animal products and an increase in the intake of plant-based products (49). Consistent with these findings, a previous study pointed out that a plant-based diet showed a reduction in visceral adipose tissue (50). A high GHGEs diet typically consists of frequent consumption of red and processed meats, dairy products, and energy-dense, ultra-processed foods (8, 44, 51). In contrast, transitioning to a low GHGEs diet involves reducing these items and incorporating more plant-based foods, such as legumes, whole grains, fruits, vegetables, and nuts. These dietary patterns align closely with the principles of the Mediterranean diet, which emphasizes plant-based ingredients and minimal animal products, contributing to both environmental sustainability and improved health outcomes (18, 20, 43).

The increase of adipose tissue registered in obesity may be also related to dietary CO_2_ emissions. Previous studies showed that obesity is associated with around 20% greater GHGEs relative to the normal weight state, because of increased oxidative metabolism due to greater metabolic demands. Globally, obesity contributes to an extra around 700 megatons per year of CO_2_ equivalent, which is about 1.6% of global GHGEs (52).

It has been also pointed out that environmental factors such as diet, activity, stress, and environmental pollution could modify some genes, leading to increased body fatness (53). Environmental contaminants were also associated with metabolic disruptions, becoming a contributing factor to changes in body composition (54). Controlling some of these factors with a healthy, and sustainable diet could be a possible solution to avoid those unwanted body composition changes.

It has been estimated that a 10 kg weight loss of all obese and overweight people would result in a decrease of 49.560 Mt. of CO_2_ per year, which would equal to 0.2% of the CO_2_ emitted globally in 2007. This reduction could help meet the CO_2_ emission reduction targets and would have a great benefit to the global health (55).

Therefore, moving toward healthier lifestyles would improve the body composition and, at the same time, would alleviate the current environmental detrimental situation, which is affecting the planetary health (56–58).

### 4.1 Strengths and limitations

The current paper is a new source of information, since it allows to consider anthropometric measurements under an environmental perspective, and not only under a healthy point of view. The huge sample size of the PREDIMED-Plus trial is the very first strength of this paper. A validated food frequency questionnaire was used by experimented dietitians to record dietary intake precisely, which is the second strength. Grams consumed by each participant were summed and used to calculate GHGEs in kg of CO_2_eq, taking data from AGRIBALYSE database, which considers all the processing steps, and would be considered as the third strength. Measuring anthropometrics in duplicate represents a third strength to avoid possible measuring errors, and the use of the bioimpedance analysis is a reliable technic in research, since it is done within a specific action protocol. Body composition measurements (fat mass, muscular mass, fat free mass and total body water, measured in kg) were adjusted by height (squared size in m^2^), and data for continuous variables and UNIANOVA were calculated adjusted by sex.

This paper has some limitations too. Causal interferences cannot be established because of the cross-sectional design. Considering CO_2_ alone to evaluate sustainability is a limitation because the lack of other parameters such as energy, land, or water. Results cannot be extrapolated to a younger population since our participants were between 55 and 75 years old. Finally, fat-free mass could be better estimated independently from water, since fat-free mass hydration would be greater in older population (37).

## 5 Conclusion

Body composition is associated with dietary CO_2_ emissions. The higher energy intake and correspondingly higher total body water, fat free mass, and body weight, the higher the dietary CO_2_ emissions were, following a linear relationship. Identifying less environmentally harmful diets with lower GHGEs and, promoting their adoption among the population could serve as a strategy to enhance both human health and environmental sustainability.

## Data Availability

There are restrictions on the availability of the data of this trial due to the signed consent agreements around data sharing, which only allow access to external researchers for studies following the project’s purposes. Requestors wishing to access the trial data used in this study can make a request by emailing pep.tur@uib.es.

## Footnotes

^1^ http://www.isrctn.com/ISRCTN89898870
